# Targeting on the NAD^+^‐mitophagy axis to treat cardiovascular disease

**DOI:** 10.1002/agm2.12123

**Published:** 2020-09-24

**Authors:** Evandro F. Fang, Jun Tao

**Affiliations:** ^1^ Department of Clinical Molecular Biology University of Oslo and Akershus University Hospital Lørenskog Norway; ^2^ The Norwegian Centre on Healthy Ageing (NO‐Age) Oslo Norway; ^3^ Department of Hypertension and Vascular Disease the First Affiliated Hospital Sun Yat‐Sen University Guangzhou China

## 1 INTRODUCTION

In view of the recent finding of impaired NAD^+^‐mitophagy axis in the cardiovascular disease‐predisposed Werner syndrome,^1^ we here discuss the application of such discovery in general age‐predisposed cardiovascular disease.

## 2 CARDIOVASCULAR DISEASE IS A MAJOR BURDEN IN THE ELDERLY

Cardiovascular disease (CVD) is the leading cause of death worldwide, responsible for over 30% (approximately 18 million) of all deaths in 2016 (World Health Organization). Vascular dysfunction resulting from atherosclerosis, thrombosis or high blood pressure is a common cause of CVD, which compromises organ function.^2^, ^3^, ^4^ Ageing and inflammation are the principle drivers of vascular diseases, including the development and clinical manifestations of atherosclerosis. Atherosclerosis is a progressive inflammatory disease characterized by accumulation of cholesterol‐laden lipoproteins in the arterial vessel wall.^4^ The molecular mechanisms underlying atherosclerosis pathogenesis are not fully understood with current therapeutic interventions not satisfactory.

## 3 IMPAIRED NAD^+^‐MITOPHAGY AXIS LINKS TO CARDIOVASCULAR DISEASE

Mitochondrial dysfunction plays a major role in atherosclerosis etiology.^4^ Mitochondria are considered as the “powerhouses” of cells that play a critical role in developmental and adult neuroplasticity and neuronal survival.^1^, ^5^ There is a strong association between increased production of mitochondrial reactive oxygen species (ROS), accumulation of mitochondrial DNA damage, respiratory chain dysfunction and atherosclerosis.^3^, ^4^ Thus, efficient cellular clearance of damaged mitochondria through mitochondrial specific autophagy, termed mitophagy, plays a fundamental role in maintaining mitochondrial homeostasis and health. Autophagy is a unique membrane trafficking process where double‐membraned phagophores engulf damaged subcellular organelles and other components for lysosomal digestion and recycling of nutrients. Emerging evidence highlights a pivotal role of autophagic flux in maintaining normal vessel wall biology, while defective autophagy may be a common cause of vascular ageing and the development of associated pathologies.^3^ We and others have demonstrated an age‐dependent dysregulation of mitophagy, which is implicated in the etiology of accelerated ageing and neurological disorders.^1^, ^5^ However, the role of mitophagy in vascular homeostasis and atherosclerosis is poorly understood. It is unclear whether there is a cause‐consequence relationship between age‐dependent dysregulation of mitophagy, vascular malfunction and atherosclerosis.

In the conditions of normal ageing and possible pathological ageing (e.g. Werner syndrome/WS), there are impaired mitophagy (or macro‐autophagy), reduced vessel tissue‐resident endothelial stem cells (ESCs), and reduced blood flow. Each of the three impairments further induces endothelial dysfunction, inflammation, and may finally lead to atherosclerosis. Targeting on the restoration of the NAD^+^‐mitophagy axis may inhibit disease progression. Abbreviations: NAD^+^, nicotinamide adenine dinucleotide; WRN, Werner; MiDAS, mitochondrial dysfunction‐associated senescence; YAP, Yes‐associated protein; TAZ, transcriptional coactivator with PDZ‐binding motif; JNK, c‐Jun N‐terminal kinase.

Our recent findings in the roundworm *C. elegans* and fly *Drosophila* model of accelerated ageing, namely Werner syndrome (WS), as a result of mutations of the gene which encodes the DNA repair protein Werner (WRN), implicate a causative role of mitophagy dysregulation in WS, which was verified in human samples of WS.^1^ In particular, we identified accumulation of damaged mitochondria and defective mitophagy in WS due to DNA damage‐induced nicotinamide adenine dinucleotide (NAD^+^) depletion; and NAD^+^‐induced mitophagy enhances mitochondrial quality and fat metabolism that coincides with inhibition of accelerated ageing in the WS animal models.^1^ WS is an important model to study the mechanisms of age‐related metabolic and vascular diseases in view of significant clinical phenotypes of insulin‐resistant diabetes, hypertension, dyslipidemia and atherosclerosis resembling normal human ageing.^1^ Indeed, atherosclerotic vascular diseases, such as myocardial infarction, is a primary cause of death of WS patients.^6^

The mitophagy inducer NAD^+^ is a fundamental molecule necessary for all living organism.^5^ NAD^+^ levels decline in an age‐dependent manner.^1^, ^5^ It is a necessary cofactor for many key metabolic pathways, including glycolysis, tricarboxylic acid (TCA) cycle, fatty acid β‐oxidation, and mitochondrial oxidative phosphorylation (OXPHOS).^5^ Furthermore, NAD^+^ is also a coenzyme for at least three groups of proteins, including cyclic ADP‐ribose synthases, poly(ADP‐ribose) polymerases (PARPs), and deacetylases from the sirtuin family (SIRTs).^5^ With the growing interest in NAD^+^ in healthy ageing, it seems that NAD^+^ affects broad organ systems and modulates an expanding list of disease processes. We recently proposed that the NAD^+^‐mitophagy axis plays a fundamental role in maintaining healthy ageing and neuroprotection.^5^ Moreover, a recent study shows that NAD^+^ boosting inhibits age‐dependent endothelial dysfunction, leading to improvement of neovascularization, blood flow, and increased mobility and endurance of aged mice.^7^ The integrin‐YAP (Yes‐associated protein)/TAZ (transcriptional coactivator with PDZ‐binding motif)‐JNK (c‐Jun N‐terminal kinase) cascade is a newly discovered pathway which mediates atheroprotective effects of unidirectional shear flow.^2^

**Figure 1:**
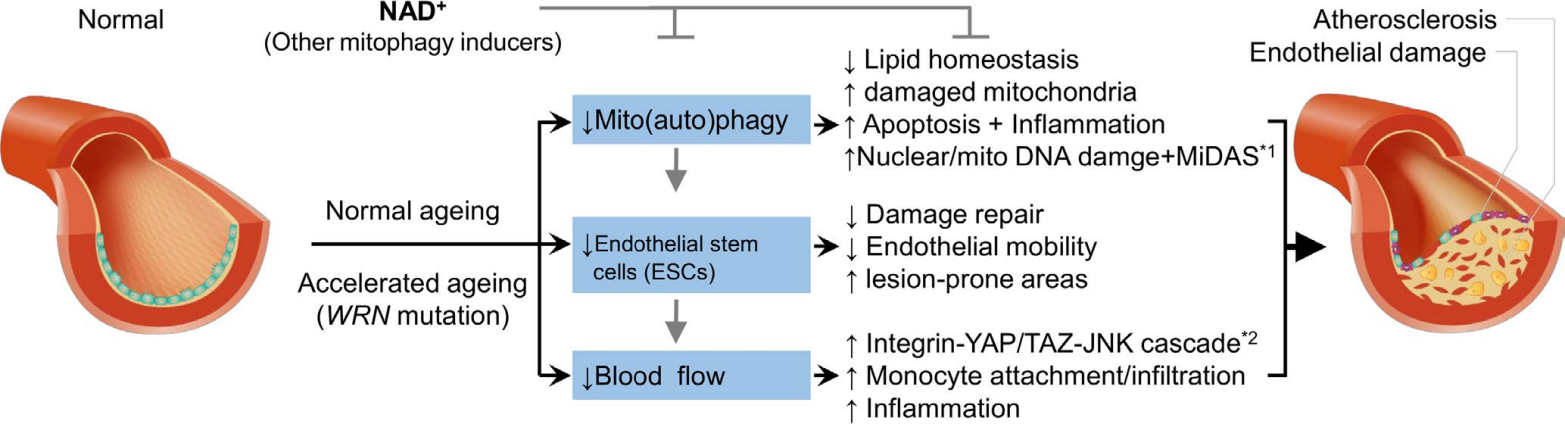
A schematic hypothesized mechanism of ageing in atherosclerosis and a possible therapeutic strategy targeting on the restoration of the NAD^+^‐mitophagy axis

Based on current literature and our unpublished data, we propose an age‐dependent impairment of mitophagy causes endothelial dysfunction, metabolic imbalance, inflammation, as well as senescence, collectively contributing to atherosclerosis; accordingly, targeting on the NAD^+^‐mitophagy pathway may provide a therapeutic strategy for CVD, including the reduction of the occurrence of atherosclerosis (Figure 1).

## 4 CONCLUSIONS AND FUTURE PERSPECTIVES

NAD^+^ maintains a broad metabolic homeostasis and protects against age‐dependent endothelial dysfunction and may provide novel preventive/therapeutic strategies in pathological and normal vascular ageing‐associated atherosclerosis. Further animal and clinical studies to verify such NAD^+^‐dependent benefits are necessary.

## CONFLICTS OF INTEREST

Nothing to disclose.

## References

[1] Fang EF , Hou Y , Lautrup S , et al. NAD+ augmentation restores mitophagy and limits accelerated aging in Werner syndrome. Nat Commun. 2019;10(1):5284. 31754102 10.1038/s41467-019-13172-8 PMC6872719

[2] Wang L , Luo JY , Li B , et al. Integrin‐YAP/TAZ‐JNK cascade mediates atheroprotective effect of unidirectional shear flow. Nature. 2016;540(7634):579–582. 27926730 10.1038/nature20602

[3] Torisu K , Singh KK , Torisu T , et al. Intact endothelial autophagy is required to maintain vascular lipid homeostasis. Aging Cell. 2016;15(1):187–191. 26780888 10.1111/acel.12423 PMC4717267

[4] Moore KJ , Sheedy FJ , Fisher EA . Macrophages in atherosclerosis: a dynamic balance. Nat Rev Immunol. 2013;13(10):709–721. 23995626 10.1038/nri3520 PMC4357520

[5] Lautrup S , Sinclair DA , Mattson MP , Fang EF . NAD+ in brain aging and neurodegenerative disorders. Cell Metab. 2019;30(4):630–655. 31577933 10.1016/j.cmet.2019.09.001 PMC6787556

[6] Takemoto M , Mori S , Kuzuya M , et al. Diagnostic criteria for Werner syndrome based on Japanese nationwide epidemiological survey. Geriatr Gerontol Int. 2013;13(2):475–481. 22817610 10.1111/j.1447-0594.2012.00913.x

[7] Das A , Huang GX , Bonkowski MS , et al. Impairment of an endothelial NAD+‐H2S signaling network is a reversible cause of vascular aging. Cell. 2018;173(1):74–89.e20. 29570999 10.1016/j.cell.2018.02.008 PMC5884172

